# Y-27632, a ROCK inhibitor, improved laser-induced shock wave (LISW)-induced cochlear synaptopathy in mice

**DOI:** 10.1186/s13041-021-00819-1

**Published:** 2021-07-03

**Authors:** Yutaka Koizumi, Kunio Mizutari, Satoko Kawauchi, Shunichi Sato, Akihiro Shiotani, Seiji Kakehata

**Affiliations:** 1 Department of Otolaryngology-Head and Neck Surgery, Yamagata University Faculty of Medicine, 2-2-2 Iida-Nishi, Yamagata, 990-9585 Japan; 2 Department of Otolaryngology-Head and Neck Surgery, National Defense Medical College, 3-2 Namiki, Tokorozawa, Saitama 359-8513 Japan; 3 Division of Bioinformation and Therapeutic Systems, National Defense Medical College Research Institute, Saitama, 359-8513 Japan

**Keywords:** Cochlea, Rho-associated coiled-coil containing protein kinase (ROCK), Synapse, Regeneration, Y-27632, Hearing loss, Inner ear

## Abstract

Recently, a pathological condition called cochlear synaptopathy has been clarified, and as a disorder of the auditory nerve synapses that occurs prior to failure of hair cells, it has been recognized as a major cause of sensorineural hearing loss. However, cochlear synaptopathy is untreatable. Inhibition of rho-associated coiled-coil containing protein kinase (ROCK), a serine-threonine protein kinase, has been reported to have neuroprotective and regenerative effects on synaptic pathways in the nervous system, including those in the inner ear. We previously demonstrated the regenerative effect of the ROCK inhibitor, Y-27632, on an excitotoxic cochlear nerve damage model in vitro. In this study, we aimed to validate the effect of ROCK inhibition on mice with cochlear synaptopathy induced by laser-induced shock wave (LISW) in vivo. After the elevation of ROCK1/2 expression in the damaged cochlea was confirmed, we administered Y-27632 locally via the middle ear. The amplitude of wave I in the auditory brainstem response and the number of synapses in the Y-27632-treated cochlea increased significantly. These results clearly demonstrate that ROCK inhibition has a promising clinical application in the treatment of cochlear synaptopathy, which is the major pathology of sensorineural hearing loss.

## Introduction

Primary cochlear neural degeneration, which is also called cochlear synaptopathy, is recognized as a common pathology associated with sensorineural hearing loss [1], which is caused by various etiologies, such as noise [2], aging [3], congenital genetic factors [4], and blast exposure [5]. A unique characteristic of this pathology is that it is not accompanied by the loss of hair cells, which are the primary receptors of sound. The clinical characteristic of this pathology is also unique in that it causes mild or sometimes no hearing threshold shift, but it is closely related to the pathogenesis of tinnitus [6, 7] and hyperacusis [8]. Cochlear synaptopathy, commonly referred to as “hidden hearing loss,” has now been recognized as the critical therapeutic target because quite a few proportions of patients with hearing impairment suffer from this pathology, and it is still untreatable [9, 10].

Rho-associated coiled-coil containing protein kinase (ROCK), a serine-threonine protein kinase, is a target protein of small molecular weight GTP-binding protein Ras homolog (Rho) [11], and activation of the ROCK pathway has been associated with inhibition of neurite regeneration and outgrowth in patients with spinal cord injury [12]. In contrast, inhibition of the Rho/ROCK pathway has an effect on axonal regeneration in the peripheral nerves [13, 14], including the cochlear nerve [15, 16]. In our previous study, we examined the effects of ROCK inhibitors on the damaged auditory nerve end and synapses using an excitotoxic cochlear organotypic model and found that ROCK inhibitors could regenerate the cochlear nerve axons and synapses between the inner hair cell (IHC) and the auditory nerve after excitotoxic injury of the cochlea [17]. Based on these findings, we hypothesized that ROCK inhibitors would exert their effects on synaptic remodeling even in an in vivo model of cochlear synaptopathy.

To validate this hypothesis, we examined the effects of ROCK inhibitors on the damaged synapse between IHC and the auditory nerve using a cochlear synaptopathy model generated by a laser-induced shock wave (LISW). The LISW-induced hearing dysfunction model was developed as a blast-induced hearing loss model that replicates a blast-exposed ear with pure sensorineural hearing loss without any conductive hearing loss [5]. The greatest advantage of this animal model is that LISW can reduce the number of synapses without any hair cell loss, which is the most characteristic pathology of cochlear synaptopathy, with good reproducibility. We examined the changes in ROCK expression in the cochlea with synaptopathy and then administered a ROCK inhibitor to the damaged cochlea locally. Herein, we report the therapeutic effect of a ROCK inhibitor on cochlear synaptopathy in vivo.

## Results

### ROCK1 and ROCK2 expression in the organ of Corti after LISW-induced cochlear damage

In this study, we used a mouse model of cochlear synaptopathy induced by direct LISW exposure of the inner ear. The output level of LISW was set to induce cochlear synaptopathy, which is associated with synaptic degeneration without hair cell loss, according to a previous study [5], and we then reproduced synaptic loss in mice without both inner and outer hair cell loss.

**Fig. 1:**
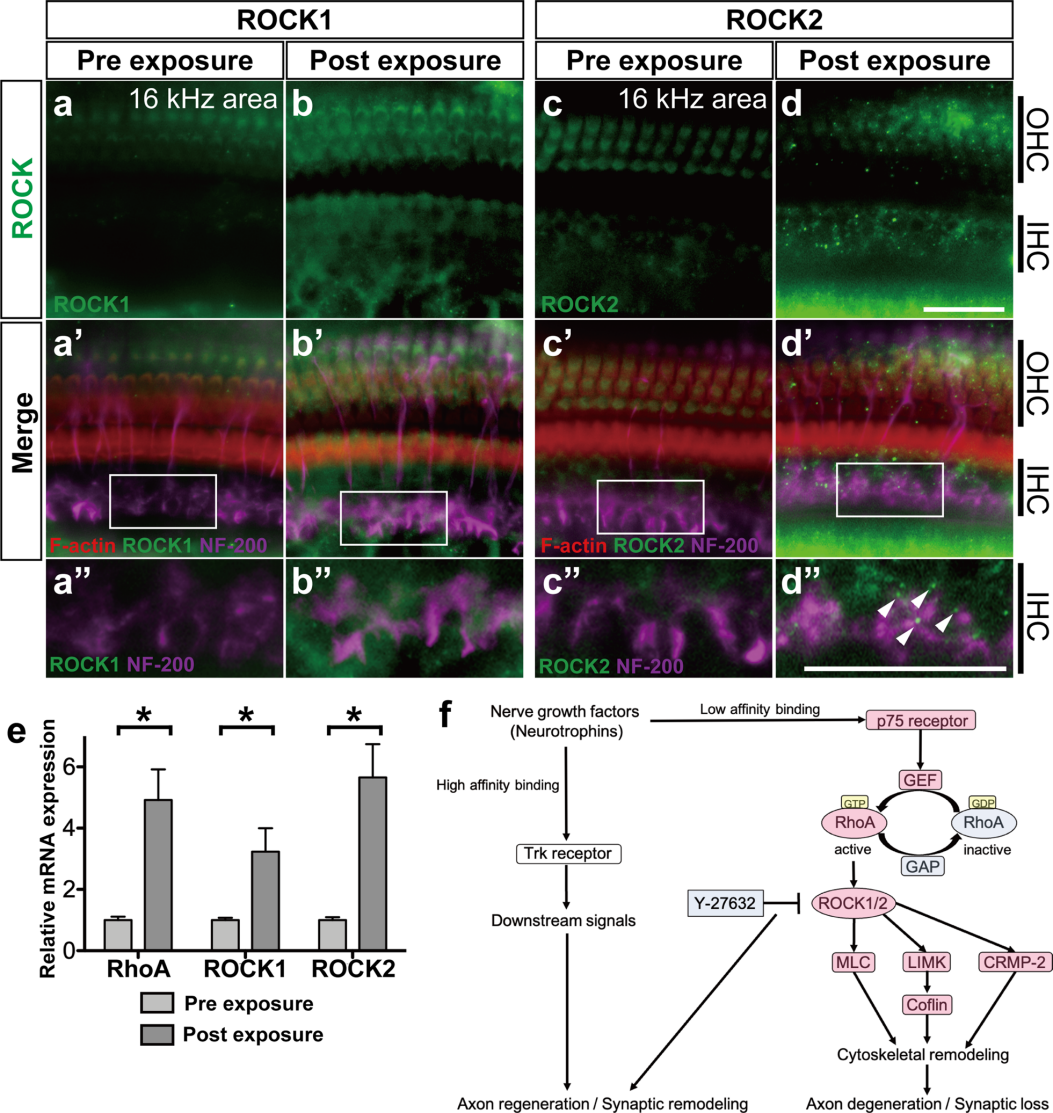
Changes in the expression levels of ROCKs before and after cochlear damage induced by irradiation of the organ of Corti with laser-induced shock wave (LISW). (**a**–**d**, **a**’–**d**’, **a**”–**d**”) Projection images of a confocal series of immunohistochemistry: tissues of the cochlear organ of Corti from the middle turn (16 kHz area) of the mouse before and 1 day after LISW exposure. Immunohistochemistry for ROCK1 (green, anti-ROCK1 (**a**, **b**), anti-ROCK2 (**c**, **d**)) and merged images with F-actin (rhodamine phalloidin, red) and neurofilament (NF-200, purple) are shown in a’-d’. (**a**”–**d**”) Enlarged images of the white inlets in (**a**’–**d**’) focusing on the inner hair cells and the end of the peripheral axons of the auditory nerve. After LISW exposure, ROCK1 expression showed ubiquitous elevation around the nerve end (**b**”), whereas ROCK2 expression showed punctiform enhancement around the inner hair cells (**d**”, white arrowheads) in addition to the ubiquitous elevation of expression. **e** The relative mRNA expression levels of RhoA, ROCK1, and ROCK2 before and 1 day after LISW exposure. The relative mRNA expression levels were standardized by the expression levels before LISW exposure in each mRNA (n = 6). **f** The schema of Rho/ROCK and associated signal pathway. *indicates a significant difference (*p* < 0.05, two-tailed Mann–Whitney *U* test). The data are shown as the mean ± standard errors of mean. *CRMP-2* collapsing response mediator protein-2, *GAP* GTPase-activating protein, *GEF* guanine nucleotide-exchange factor, *IHC* inner hair cell, *LIMK* LIM kinase, *MLC* myosin light chain, *OHC* outer hair cell, *Trk* tropomyosin receptor kinase. Scale bar: 20 µm.

First, we examined the expression of ROCK1 and ROCK2 in the cochlea before and 1 day after the local administration of 10 mM Y-27632 to the cochlea (2 days after LISW exposure) because these expression patterns seemed to change immediately on the first day after excitotoxic injury in our previous study. In the native cochlea, ROCK1 was expressed around the outer hair cell area; however, no expression was observed around the IHC area (Fig. 1a, a’, a”). After LISW exposure, ROCK1 expression was obviously elevated in both the inner and outer hair cell areas (Fig. 1b, b’). ROCK1 expression was likely seen in outer hair cells, Deiters’ cells, and inner hair cells except in the pillar cells. Around the IHC area, elevated ROCK1 expression was observed ubiquitously and partly with punctiform pattern around the end of the NF200-positive peripheral axons in the inner spiral sulcus area (Fig. 1b’, b”, around the purple area). Additionally, elevation of ROCK1 expression was observed in the cytoplasm of the outer hair cells (Fig. 1b). The expression pattern of ROCK2 in the native cochlea was similar to that of ROCK1, and weak expression was observed in the outer hair cell area. After cochlear damage, ROCK2 expression was increased at the end of the NF200-positive peripheral axon area as seen in the outer hair cell area (Fig. 1d, d’, d’’). The characteristic punctiform elevation pattern of ROCK2 was observed (Fig. 1d, white arrowheads).

We subsequently confirmed the changes in mRNA expression of ROCK1, ROCK2, and RhoA, which is an upstream effector of ROCKs in the Rho/ROCK pathway 1 day after cochlear damage. Quantitative polymerase chain reaction (PCR) revealed that the mRNA expression levels of RhoA, ROCK1, and ROCK2 increased significantly after LISW exposure (4.923-fold, *p* = 0.013; 3.235-fold, *p* = 0.031; and 5.656-fold, *p* = 0.045, respectively, two-tailed Mann–Whitney U test) (Fig. 1e).

### Effect of Y-27632 on the degenerated synapse induced by LISW exposure

**Fig. 2:**
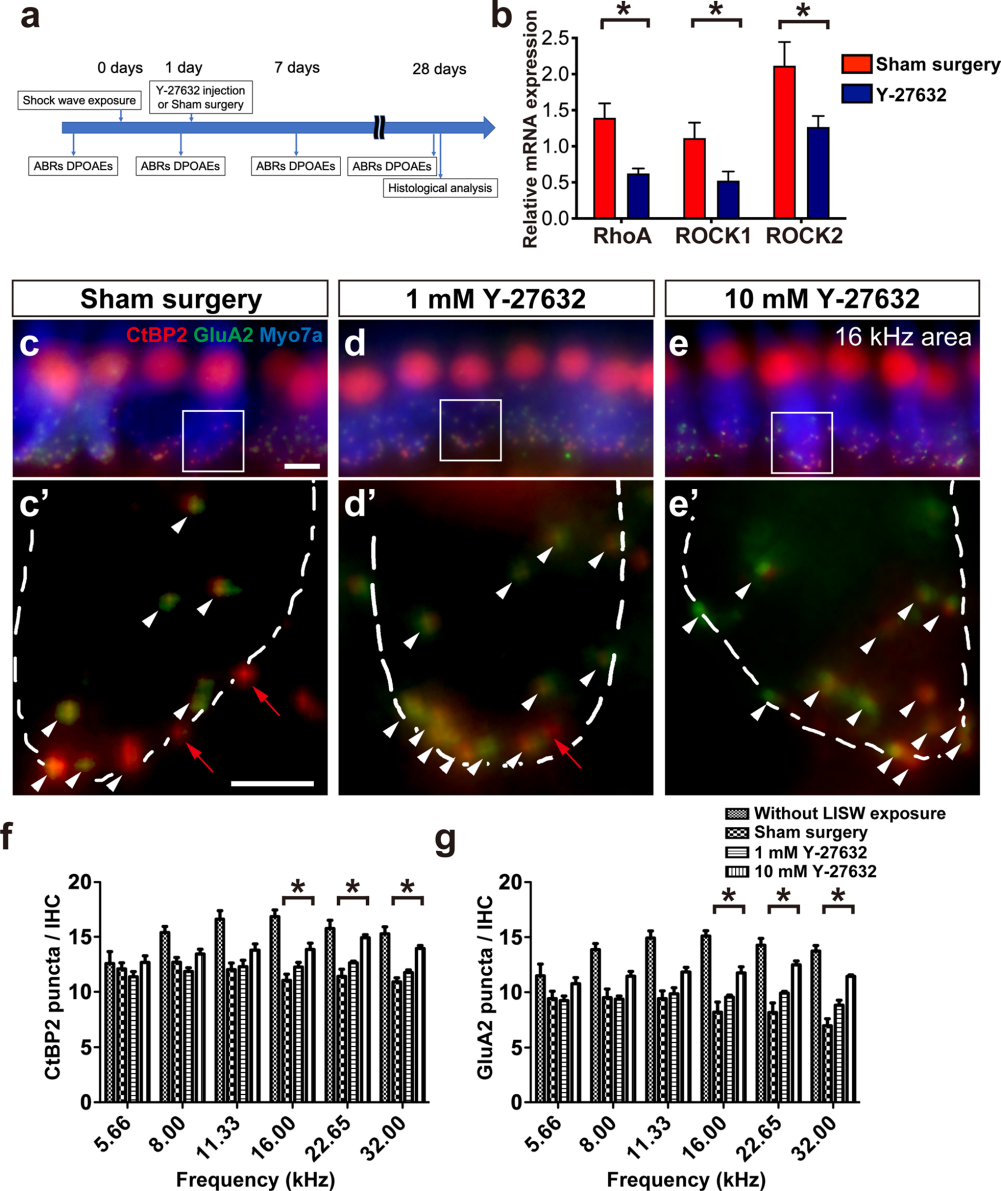
Effect of Y-27632 on the synapses in the inner hair cells after LISW exposure. **a** Timeline of the experiments in this study. **b** The effect of 10 mM Y-27632 in the LISW exposed cochleae on relative mRNA expression levels of RhoA, ROCK1, and ROCK2, 2 days after Y-27632 treatment. The relative mRNA expression levels were standardized by the expression levels before LISW exposure in each mRNA (n = 6). *indicates a significant difference (*p* < 0.05, two-tailed Mann–Whitney U test). **c**–**e** The effect of Y-27632 on the degenerated synapses of the inner hair cells at cochlear 16 kHz area 28 days after treatment with various concentrations of Y-27632 (**c** without Y-27632, d: 1 mM, and e: 10 mM). In **c**–**e**, presynaptic ribbons (CTBP2-immunoreactive puncta, red), postsynaptic densities (GluA2 immunoreactive puncta, green), and hair cells labeled with myosin 7a (blue) are shown. (**c**'–**e**') Enlarged images of the white inlets in (**c**–**e**) focusing on the synapses in the inner hair cells area. White dotted lines show the contour of the inner hair cells. White arrowhead indicates normal synapse formation with CtBP2-positive patch (red) accompanied with glutamate-receptor patch (green). Red arrow indicates orphan ribbons, which is a CtBP2-positive patch without apposed glutamate-receptor patches. In **c**’, there are seven normal synapses (white arrowheads) and two orphan synapses (red arrows); in **d**’, there are 12 normal synapses and one orphan synapse; and in e’, all 14 synapses have both CtBP2 and glutamate-receptor patch. **f**, **g** The quantification of the synaptic ribbons (**f**) and the glutamate receptor (**g**) observed in the 28 days after treatment and control from single IHC. The number of synapse components is lower in the sham surgery groups than in the control group at all frequencies tested. The number of synapse components is significantly larger in the 10 mM Y-27632-treated group at higher frequencies (asterisks) than those in the sham surgery group. Scale bar is 5 µm. *indicates significant differences (*p* < 0.05, two-way ANOVA, followed by Bonferroni correction for multiple comparisons). Values are represented as mean ± SEM

After we confirmed the upregulation of the Rho/ROCK pathway induced by cochlear damage, we used the ROCK inhibitor, Y-27632, to rescue cochlear synaptopathy after LISW exposure. First, we measured the mRNA expression of RhoA, ROCK1, and ROCK2 2 days after the local administration of 10 mM Y-27632 to the cochlea. All of them were significantly more suppressed by local Y-27632 treatment than by sham surgery (RhoA: 0.448 folds, *p* = 0.006; ROCK1: 0.472, *p* = 0.0463; ROCK2: 0.600 folds, *p* = 0.049, two-tailed Mann–Whitney U test; Fig. 2b).

Next, we measured synaptic markers in the inner hair cells, including presynaptic ribbons stained by CtBP2 and postsynaptic puncta labeled by GluA2 to explore the functional connection between the IHC and auditory peripheral axons 1 month after local treatment with Y-27632. The numbers of CtBP2 puncta and GluA2 puncta in the sham surgery group were significantly lower than those in the control group (without LISW exposure) at all frequencies, except at 5.6 kHz (CtBP2: two-way ANOVA, F_5, 120_ = 4.344, *p* = 0.001; Bonferroni multiple comparison significance at 8.0 kHz [*p* = 0.005], at 11.3 kHz [*p* < 0.001], at 16.0 kHz [*p* < 0.001], at 22.6 kHz [*p* < 0.001], at 32.0 kHz [*p* < 0.001]; Fig. 2f) (GluA2: two-way ANOVA, F_5, 120_ = 3.373, *p* = 0.007; Bonferroni multiple comparison significance at 8.0 kHz [*p* < 0.001], at 11.3 kHz [*p* < 0.001], at 16.0 kHz [*p* < 0.001], at 22.6 kHz [*p* < 0.001], at 32.0 kHz [*p* < 0.001]; Fig. 2g). Enlarged images focusing on the synapses in the inner hair cell area (Fig. 2c, c’) showed a decrease in the number of presynaptic ribbons (CtBP2-immunoreactive puncta, red) and also confirmed the presence of orphan synapses (Fig. 2c, c’, red arrows), which is the synaptic structure lacking apposed glutamate-receptor patches (GluA2 immunoreactive puncta, green). In the 1 mM Y-27632-treated group, the numbers of CtBP2 puncta and GluA2 puncta were still lower at any frequency than those in the sham surgery group; however, the trend of increase in both puncta was observed (CtBP2: Bonferroni multiple comparison significance at 5.6 kHz [*p* > 0.999], 8.0 kHz [*p* > 0.999], at 11.3 kHz [*p* > 0.999], at 16.0 kHz [*p* = 0.752], at 22.6 kHz [*p* = 0.719], at 32.0 kHz [*p* > 0.999]; Fig. 2d, d’, f) (GluA2: Bonferroni multiple comparison significance at 5.6 kHz [*p* > 0.999], 8.0 kHz [*p* > 0.999], at 11.3 kHz [*p* > 0.999], at 16.0 kHz [*p* = 0.592], at 22.6 kHz [*p* = 0.207], at 32.0 kHz [*p* = 0.168]; Fig. 2d, d’, g). Otherwise, the numbers of CtBP2 puncta and GluA2 puncta in the 10 mM Y-27632 group increased significantly at higher frequencies than those in the sham surgery group (CtBP2: Bonferroni multiple comparison significance at 16.0 kHz [*p* = 0.003], at 22.6 kHz [*p* < 0.001], at 32.0 kHz [*p* = 0.001]; Fig. 2e, e’, f) (GluA2: Bonferroni multiple comparison significance at 16.0 kHz [*p* < 0.001], at 22.6 kHz [*p* < 0.001], at 32.0 kHz [*p* < 0.001]; Fig. 2e, e’, g). These results suggest that Y-27632 has a dose-dependent effect on synaptic regeneration in LISW-induced cochlear synaptopathy.

### Effect of Y-27632 on the sensorineural hearing dysfunction after LISW exposure

**Fig. 3:**
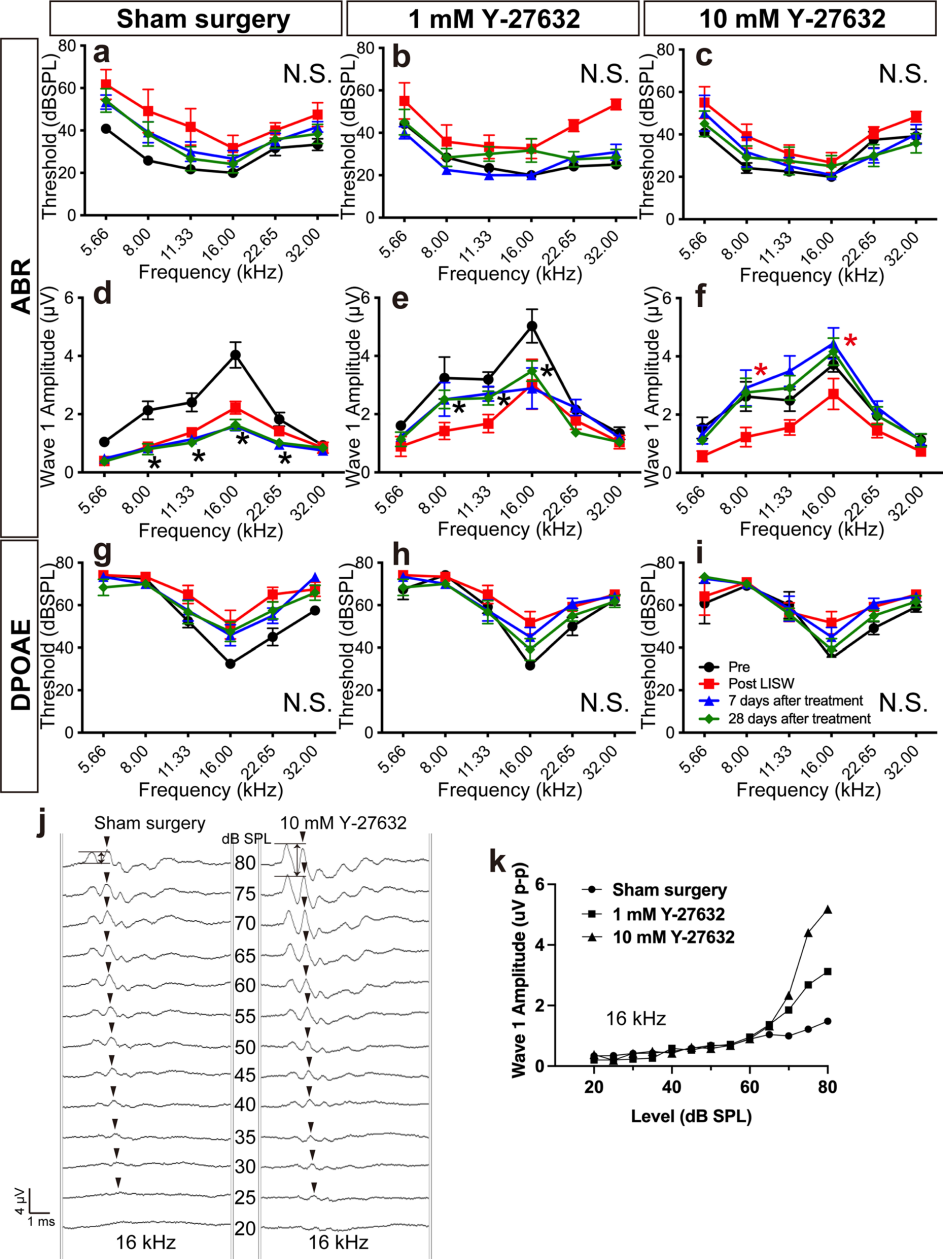
Measurement of hearing function using ABR and DPOAE before and after Y-27632 treatment. **a**–**c** Changes in ABR threshold at various timepoints are shown in Fig. 2a. The ABR thresholds at 28 days after Y-27632 treatment (green filled diamonds) are not significantly different from those at pre-LISW exposure (black filled circle) in any treatment groups, although the temporal threshold shifts at 1 day after LISW exposure can be observed. **d**–**f** ABR wave I amplitudes at the same timepoints as in a-c. Similar to the ABR threshold changes, a decrease in temporal amplitude can be observed at 1 day after LISW exposure in any groups. However, in the 1 mM Y-27632-treated group, the amplitude at 28 days after treatment (green filled diamonds) from 8 to 16 kHz is still significantly (black asterisks) lower than that at pre-LISW exposure (black filled circle). In 10 mM Y-27632 treatment group, the amplitude at 28 days after treatment (green filled diamonds) at any frequencies is not significantly different from that at pre-LISW exposure (black filled circle). However, the amplitudes at 28 days after treatment (green filled diamonds) at 8 kHz and 16 kHz are significantly larger than those at post-LISW exposure (red filled squares). **g**–**i** Changes in DPOAE threshold at various timepoints are shown in Fig. 2a. No significant elevations can be observed in the DPOAE thresholds in any treatment group. **j** An example of 16 kHz ABR waves recorded at 28 days after Sham surgery and 10 mM Y-27632 treatment. Bilateral arrows show the wave I amplitude at 80 dB SPL. Arrowheads show the peaks with the largest peak-to-peak amplitude. In the Y-27632-treated ear, the peak could first be detected at 25 dB, while on the control side, the peak could first be detected at almost the same sound pressure level. **k** The representative figure of the input–output curve recorded at 28 days after Sham surgery (filled circle), 1 mM Y-27632 treatment (filled square), and 10 mM Y-27632 treatment (filled triangle). Black asterisk indicates significant changes in the value at 28 days after treatment than at pre-LISW exposure (*p* < 0.05, two-way ANOVA, followed by Bonferroni correction for multiple comparisons). Red asterisk indicates significant changes in the value at 28 days after treatment than at post-LISW exposure (*p* < 0.05, two-way ANOVA, followed by Bonferroni correction for multiple comparisons). Values are represented as mean ± SEM

Finally, we conducted the cochlear function test, which includes auditory brainstem responses (ABRs) and distortion product otoacoustic emissions (DPOAEs), before and after exposure to LISW and the ROCK inhibitor, Y-27632, at several time points (Fig. 3). In the sham surgery group, the ABR and DPOAE thresholds were temporarily elevated immediately after LISW exposure; however, the threshold was recovered 7 days after sham surgery with no significant difference at all frequencies tested (Fig. 3a, g). Although the elevation of ABR threshold was recovered, the ABR wave I amplitude decreased from 1 day after LISW exposure and continued to decrease up to 28 days after sham surgery with significant differences from 8.00 kHz to 22.65 kHz (two-way ANOVA, F_5, 120_ = 46.20, *p* = 0.007; Bonferroni multiple comparison significance at 8.00 kHz [*p* < 0.001], at 11.33 kHz [*p* < 0.001], at 16.00 kHz [*p* < 0.001], at 22.65 kHz [*p* = 0.014]; Fig. 3d, black asterisks). These results clearly showed that LISW exposure in this research setting could induce pure cochlear synaptopathy, which is the pathology of ABR wave I amplitude elevation without ABR/DPOAE threshold elevation.

Similar to the sham surgery group, the 1 mM Y-27632-treated group did not show a permanent ABR/DPOAE threshold shift (Fig. 3b, h). In addition, the ABR wave I amplitude at 28 days after 1 mM Y-27632 treatment continued to decrease and was significantly different from that before LISW exposure (two-way ANOVA, F_5, 120_ = 23.13, *p* < 0.001); however, this parameter at 22.65 kHz was not significantly different from that before LISW exposure at the same frequency (Bonferroni multiple comparison significance at 22.65 kHz [*p* = 0.916]; Fig. 3e). Interestingly, the ABR wave I amplitude with 10 mM Y-27632 treatment started to improve 7 days after treatment. This improvement of ABR wave I amplitude continued until 28 days after treatment and was significantly different from that after LISW exposure at 8.00 and 16.00 kHz at the 28th day (two-way ANOVA, F_5, 120_ = 23.13, *p* < 0.001; Bonferroni multiple comparison significance at 8.00 kHz [*p* = 0.021], and 16.00 kHz [*p* = 0.031]; Fig. 3f, red asterisks). The ABR/DPOAE threshold did not significantly change at 28 days after treatment as well as in the sham surgery or 1 mM Y-27632 treatment groups (ABR: two-way ANOVA, F_5, 120_ = 15.08, Bonferroni multiple comparison significance at all frequencies [*p* > 0.999]; Fig. 3c) (DPOAE: two-way ANOVA, F_5, 120_ = 22.68, Bonferroni multiple comparison significance at all frequencies [*p* > 0.999]; Fig. 3i). These results also suggest that the ROCK inhibitor, Y-27632, has a dose-dependent effect on the recovery of hearing function in cochlear synaptopathy.

## Discussion

In this study, we showed the involvement of the Rho/ROCK pathway in the synapse degeneration and recovery effect of a ROCK inhibitor on cochlear synaptopathy induced by LISW exposure. We observed an increase in mRNA expression levels of RhoA, ROCK1, and ROCK2 in the cochlea immediately after LISW exposure and localized expression of ROCKs in the organ of Corti. Although the increase in mRNA expression levels was transient, the changes in mRNA expression levels and localized expression of ROCKs were similar to the results of our previous in vitro study that used the excitotoxic cochlear damage model [17]. We also found that the local administration of the ROCK inhibitor, Y-27632, to the inner ear could promote synaptic reorganization and recover hearing function, as confirmed by a re-increase in ABR wave I amplitude. ROCK inhibitor was administered 24 h after LISW exposure, and recovery of cochlear function may occur within 1 week. There were several reports of the Rho signaling pathway on the synaptic damage, which suspected that the pathway activation and synaptic remodeling rapidly occurred immediately after the synaptic damage. The RhoA pathway activation and synaptic disruption occur within several hours after retinal injury [18] or sevoflurane exposed hippocampal neurons [19]. On the other hand, the onset of action by ROCK inhibitors was also shown to be rapid [18–20]; therefore, the results of this study are considered to be consistent with these reports. Unlike our previous study, this study showed an increase of presynaptic marker CtBP2 as well as postsynaptic marker GluA2. Since the excitotoxic model in vitro does not cause the disappearance of presynaptic marker [21], the effect of ROCK inhibitors was only seen in postsynaptic marker. However, both presynaptic and postsynaptic markers are reduced in the LISW model [5]; thus, the effects of ROCK inhibitors on both presynaptic and postsynaptic markers could be seen. In this study, we adopted local administration of ROCK inhibitor onto the round window membrane via surgery to deliver the drug into the cochlea. Drug delivery via the middle ear is an established method in animal studies [22] and clinical settings [23]. This is the first report to prove that a ROCK inhibitor could recover synapses between IHC and the auditory nerve and restore hearing function in an in vivo model of cochlear synaptopathy.

In this study, we used the LISW mouse model to induce the pathology of cochlear synaptopathy; however, conventionally, acute cochlear synaptopathy is induced by noise exposure in animal models. Using noise-induced cochlear synaptopathy, the damaged area, where synapses decrease, is generally limited in the cochlear basal turn [2, 24]. The greatest advantage of the LISW-induced cochlear synaptopathy model is that the synapse decreased area without hair cell loss is wider toward the lower frequency area [5]. As shown in Figs. 2 and 3, our model showed synaptic loss and ABR wave I amplitude decrease in the entire cochlear area. Due to this characteristic, we analyzed the effect of treatment in the cochlear area in detail. In addition, as shown in Fig. 1, the pattern of ROCK expression in the organ of Corti in the LISW model was similar to that in the excitotoxic synapse and neuron damage model in vitro, which showed that elevated ROCK1 expression was observed ubiquitously around the end of peripheral axons, and ROCK2 expression was increased at the end of the axon area as a punctiform pattern [17]. Cochlear excitotoxicity has been hypothesized to represent a critical aspect of cochlear synaptopathy [25]. Therefore, our mouse model is an ideal platform for developing new treatment strategies for cochlear synaptopathy.

Rho/ROCK pathway has a specific action in the remodeling of actin [26] and plays important roles in morphogenesis, migration, proliferation, and cell survival [27] (Fig. 1f). Rho-GTPases cycle between an active GTP-bound state and an inactive GDP-bound state, and these molecular states are switched by guanine nucleotide-exchange factors (GEFs), which activate Rho-GTPases by accelerating GTP-GDP exchange, and GTPase-activating proteins (GAPs), which inhibit Rho-GTPases by GTP hydrolysis reaction [28, 29]. ROCK1/2, the major downstream effector of Rho-GTPase, interacts with active Rho-GTP bound state and activates intracellular signaling cascades. ROCK increases the phosphorylation of myosin light chain (MLC), LIM kinase (LIMK), and collapsing response mediator protein-2 (CRMP-2). MLC, which is a subunit of the actin-based motor protein myosin II, is crucial for the generation of contractile force, and phosphorylation of MLC promotes actomyosin contraction [30]. Phosphorylation of LIMK promotes the phosphorylation and inactivation of cofilin, which mediates actin turnover, resulting in actin stabilization [31]. CRMP-2 is crucial for axon growth by regulating microtubule dynamics, and phosphorylation of CRMP-2 inhibits microtubule assembly [32]. ROCK is activated by switching its conformation without phosphorylation, and ROCK inhibitors act by inhibiting this conformation change of ROCK [33]. Therefore, ROCK inhibitors may not directly affect the expression levels of RhoA, ROCK1, and ROCK2; however, these expression levels were decreased after administration of ROCK inhibitor, Y-27632. These changes in mRNA expression levels were seen in our previous study, but the detailed mechanism is unknown. Joshi et al. reported similar mRNA expression change in the peripheral motor neuron regenerated by ROCK inhibitors [13]. They explained that the regenerative ability may depend on their responsiveness to the ROCK inhibitors.

Since the pathology of cochlear synaptopathy has been clarified in recent years, there have been several reports on treatment based on this pathology, that is, protection or regeneration of IHC-auditory nerve synapses. Neurotrophic factors, such as brain-derived neurotrophic factor (BDNF) [34], neurotrophin-3 (NT-3) [21, 35], and growth factors (insulin-like growth factor 1 (IGF1) [36] have been studied for a relatively long time and shown to be effective for the treatment of hearing loss [37]; however, they are difficult to use because they show insufficient permeability at the blood–brain and blood-nerve barriers [38]. The tropomyosin receptor kinase (Trk) receptor is a high-affinity receptor for neurotrophic factors. TrkA binds to nerve growth factor (NGF) [39], BDNF, and neurotrophin-4/5 (NT-4/5) [40, 41], and TrkC binds to NT-3 [42]. Small-molecule TrkB receptor agonists have the ability to restore noise-induced hidden hearing loss, similar to its ligand, BDNF [43]. ROCK inhibitors are involved in a cascade of neurotrophic factors. The ROCK pathway is downstream of these neurotrophins via p75 receptors, a low-affinity receptor for neurotrophic factors [44, 45]. Therefore, ROCK inhibition may be a direct mechanism for synapse regeneration in cochlear synaptopathy. In addition, most candidate compounds have not been proven to be safe for use in clinical settings. Therefore, it would be easier to apply a ROCK inhibitor for neuronal regeneration in the clinical setting because Y-27632 is already in clinical use. Our results demonstrate a possible breakthrough therapeutic strategy for hearing impairment accompanied by primary synaptic degeneration.

## Methods

### Animals

Forty-five CBA/J mice (male, 6 weeks old) weighing 17–20 g were purchased from the Japan Charles River (Yokohama, Japan). The animals were provided free access to water and were fed a regular diet. Moreover, they were individually housed and maintained at 23–25 °C. In this study, all procedures on mice were performed under general anesthesia induced by intramuscular injection of ketamine (75 mg/kg) and medetomidine (1 mg/kg). All experimental procedures reported herein were approved by the Institutional Animal Care and Use Committee of the National Defense Medical College and were performed in accordance with the guidelines of the National Institutes of Health and the Ministry of Education, Culture, Sports, Science and Technology of Japan (approval #18050). All efforts were made to minimize the number of animals used and their suffering.

### LISW irradiation of the inner ear

LISWs were generated as described previously [5]: a laser target was irradiated with a 532-nm Q-switched neodymium-doped yttrium aluminum garnet (Nd: YAG) laser (Brilliant b, Quintal, Les Ulis Cerdex, France; pulse width, 6 ns). Specifically, the laser target was a 10-mm in diameter, 0.5-mm thick black natural rubber disk, and a 1.0 mm thick transparent polyethylene terephthalate sheet was bonded to the top of the target area to confine the laser-induced plasma, by which the LISW impulse was increased. The laser pulse was focused to a 4.0-mm diameter spot on the laser target using a plano-convex lens. The laser fluence on the target was set at 2.0 J/cm^2^, as described in a previous report [5], to generate the cochlear synaptopathy model without hair cell loss. Temporal pressure waveforms of LISWs were measured with a hydrophone (HNR-1000, Onda Co., Sunnyvale, CA, USA). The signals of the hydrophone were recorded using a digital oscilloscope (DPO4104B, Tektronix, Tokyo, Japan; bandwidth, 1 GHz), and they were calibrated using software provided by the manufacturer of the pressure sensor.

### Administration of Y-27632, a ROCK inhibitor, to the inner ear

Y-27632 (257-00511, Wako Pure Chemical Industries Ltd, Osaka, Japan) was dissolved in water, and the concentration was adjusted to 1 and 10 mM. PBS was used as the sham control. The following three groups were compared to investigate the effects of the ROCK inhibitor, Y-27632, on the LISW-induced cochlear neuropathic model: 1. sham surgery group (treated with PBS); 2. ROCK inhibition group (1 mM Y-27632); and 3. ROCK inhibition group (10 mM Y-27632). To administer Y-27632 to the inner ear, the left postauricular region of the mice was positioned under a stereomicroscope. After a 20-mm postauricular skin incision was made, subcutaneous tissues and superficial fascia were dissected, and the otic bulla was exposed. Tympanotomy was performed using microforceps, and the hole was enlarged to allow clear observation of the round window niche. Then, 1 µL of PBS or ROCK inhibitor was injected into the round window niche at a rate of 20 nL/sec using a Nanoject III Programmable Nanoliter injector (3-000-207, Drummond Scientific Company, Broomall, PA, USA).

### Cochlear function tests

Cochlear function tests were performed in each animal at six log-spaced frequencies (half-octave steps from 5.6 to 32.0 kHz) before and 1 day, 7 days, and 28 days after blast exposure. Mice were anesthetized with ketamine (75 mg/kg i.p.) and medetomidine hydrochloride (1 mg/kg i.p.). For ABRs, needle electrodes were inserted at the vertex and pinna, with the ground near the tail. ABRs were evoked with 5-ms tone pips (0.5-ms rise-fall with a cos2 onset envelope delivered at 35/s). The response was amplified, filtered, and averaged using a LabVIEW-driven data-acquisition system. The sound level was raised in 5 dB steps from ≥ 10 dB to < 80 dB SPL. At each sound level, 1024 responses were averaged (with alternating stimulus polarity). On visual inspection of stacked waveforms, the “ABR threshold” was defined as the lowest SPL level at which any wave could be detected, which usually corresponds to the level step just below that at which the peak-to-peak response amplitude rose significantly above the noise floor. When no response was observed at the highest sound level available, the threshold was designated as 5 dB greater than that level so that statistical tests could be performed. For amplitude versus level functions, the wave I peak was identified by visual inspection at each sound level, and the peak-to-peak amplitude was computed.

For the measurement of DPOAEs at 2f1–f2, the primary tones were set such that the frequency ratio (f2/f1) was 1.2 and the f2 level was 10 dB below the f1 level. For each f2/f1 primary pair, levels were swept in 5 dB steps from 20 to 80 dB SPL (for f2). At each level, both waveform and spectral averaging were used to increase the signal-to-noise ratio of the recorded ear-canal sound pressure, and the amplitude of the DPOAE at 2f1–f2 was extracted from the averaged spectra, along with the noise floor at nearby points in the spectrum. Iso-response curves were interpolated from the plots of the DPOAE amplitude versus sound level. The threshold was defined as the f1 level required to produce a DPOAE at 0 dB SPL.

### Quantitative RT-PCR

Harvested cochlear tissues were collected and stored until further analysis (Ambion, Austin, TX, USA). Total RNA was extracted using the RNeasy Mini Kit (Qiagen, Valencia, CA, USA) according to the manufacturer’s instructions. Quantitative RT-PCR was performed on a Thermal Cycler Dice Real-Time System using the One-Step SYBR PrimeScript PLUS RT-PCR Kit (RR096A; TaKaRa Bio, Shiga, Japan). Forward (F) and reverse (R) primer sequences were RhoA-F, 5′-AGCTTGTGGTAAGACATGCTTG-3′ and RhoA-R, 5′-GTGTCCCATAAAGCCAACTCTAC-3′; ROCK1-F, 5′-GACTGGGGACAGTTTTGAGAC-3′ and ROCK1-R, 5′-GGGCATCCAATCCATCCATCCAGC-3′; and ROCK2-F, 5′-TTGGTTCGTCATAAGGCATCAC-3′ and ROCK2-R, 5′-TGTTGGCAAAGGCCATAATATCT-3′. PCR cycling conditions included 40 cycles of 95 °C for 5 s and 60 °C for 30 s. Relative mRNA expression levels were determined using the ΔΔCt method with glyceraldehyde 3-phosphate dehydrogenase (GAPDH) as an internal control. All reactions were performed in duplicate. For assessment, relative mRNA expression levels were standardized to cochlear samples extracted before LISW.

### Cochlear processing and immunohistochemistry

Mice were perfused transcardially with 0.5 mL/g of lactated Ringer’s solution followed by 1 mL/g of 4% paraformaldehyde (PFA)/0.1 M phosphate buffer (PB) at room temperature. After decapitation, the cochlea was dissected out, and small holes were made at the round window, oval window, and apex of the cochlea; it was then bathed in 4% PFA/PB at 4 °C overnight. After decalcification with 0.5 M ethylenediaminetetraacetic acid (EDTA) (Decalcifying Soln. B (EDTA method); Wako Pure Chemical Industries Ltd, Osaka, Japan) for 4 days at 4 °C with shaking, each cochlea was microdissected into four pieces for whole-mount preparation. For immunostaining, cochlear pieces were blocked and permeabilized with 5% normal horse serum in PBS and 0.3% Triton X-100 for 1 h at room temperature, followed by overnight incubation at 37 °C with the following primary antibodies diluted in 1% normal horse serum with 0.3% Triton X-100. Chicken anti-NF 200 (1:500, Millipore, Bedford, MA, USA: AB5539) was used as an auditory nerve marker, rabbit anti-Myo7a (1:500, Proteus Biosciences Inc. Ramona, CA, USA: 25–6790) as a hair cell marker, mouse (IgG1) anti-CtBP2 (1: 500 BD Biosciences, San Jose, CA, USA: 612,044) as a presynaptic marker, mouse (IgG2a) anti-GluA2 (1: 2000 Millipore, Bedford, MA, USA: MAB397) as a postsynaptic marker, and rabbit anti-ROCK1 (1:100 abcam, Cambridge, UK: ab134181) and rabbit anti-ROCK2 (1:100 abcam: ab125025). After washing with PBS, the following secondary antibodies diluted 500-fold were incubated at room temperature for 2 h: Alexa Fluor 350-conjugated goat anti-rabbit IgG (Invitrogen, Carlsbad, CA, USA: A21068), Alexa 488-conjugated goat anti-mouse IgG2a ( A21131; Invitrogen), Alexa 488-conjugated goat anti-rabbit IgG (Invitrogen, A11034), Alexa 568-conjugated goat anti-mouse IgG1 (Invitrogen: A21124), Alexa 647-conjugated goat anti-chicken IgY (Invitrogen: A21449), and rhodamine phalloidin (1:500 Invitrogen: R415). After washing with PBS, samples were mounted with fluorescence mounting media (Dako, Santa Clara, CA, USA: S3023) and observed.

### Quantitative analysis of synapses and hair cells

The fluorescent-labeled organ of Corti was observed using a BZ-X700 fluorescence microscope (Keyence Corporation, Osaka, Japan) with a water-immersion 60 × objective and 3 × digital zoom. Optical sections in the x–y plane (z- sections) were recorded at 0.2 μm intervals along the z-axis. The resulting confocal image series (z-stack) contained a three-dimensional image of the entire volume of the cochlear tissues. The z-stack was reconstructed (to view a plane perpendicular to the x–y plane) using ImageJ (https://imagej.nih.gov/ij/) or PhotoShop CC (Adobe, San Jose, CA, USA).

To count the number of outer hair cells (OHCs) and IHCs, confocal microscopy was performed at 5.6, 8.0, 11.3, 16.0, 22.6, and 32.0 kHz while focusing on the presynaptic ribbons in the basolateral portion of IHCs; an oil-immersion 100 × or water-immersion 60 × objective and a 0.2-μm z-step were used. For each frequency region, z-stacks were acquired in each cochlea at three adjacent areas, each containing ~ 10 IHCs in a row. The number of OHCs and IHCs per 200 µm was counted at each point, as described above. The densities of OHCs and IHCs per 200 µm were calculated and compared at each site. The numbers of IHC synaptic ribbons (CtBP2-positive puncta) and glutamate-receptor patches (GluA2 puncta) per 200 µm were counted at 5.6, 8, 11.3, 16, 22.6, and 32 kHz, as described above. The number of synaptic ribbons and glutamate-receptor patches per IHC was calculated and compared at each site. To minimize bias, the counts were performed by three different individuals who were blinded to the experimental groups.

### Statistical analysis

Statistical analyses were conducted using Prism software (version 7.0; GraphPad software, Inc., La Jolla, CA, USA). For analyses of histological and cochlear function results, two-way analysis of variance (ANOVA), followed by Bonferroni correction for multiple comparisons, was used. The two-tailed Mann–Whitney U test was employed to compare differences in mRNA expression. The statistical power and the sample size were determined before and after data collection using PS: Power and Sample Size Calculation, Ver. 3.1.6 (Department of Biostatistics, Vanderbilt University, Nashville, TN, USA). Statistical significance was set at *p* < 0.05. Error bars represent standard deviation of the mean (SEM).

## Data Availability

The datasets used and/or analyzed during the current study are available from the corresponding author on reasonable request.

## Abbreviations

LISW: Laser-induced shock wave
ROCK: Rho-associated coiled-coil containing protein kinase
IHC: Inner hair cell
ABR: Auditory brainstem response
DPOAE: Distortion product otoacoustic emissions
BDNF: Brain-derived neurotrophic factor
NT-3: Neurotrophin-3
IGF1: Growth factors (insulin-like growth factor 1)
Trk: Tropomyosin receptor kinase
PFA: Paraformaldehyde
PB: Phosphate buffer
EDTA: Ethylenediaminetetraacetic acid
OHC: Outer hair cells
ANOVA: Analysis of variance
SEM: Standard deviation of the mean
CRMP-2: Collapsing response mediator protein-2
GAP: GTPase-activating protein
GEF: Guanine nucleotide-exchange factor
LIMK: LIM kinase
MLC: Myosin light chain
